# Supplementary material for: Identification of photoperiod-induced specific miRNAs in the adrenal glands of Sunite sheep (Ovis aries)
Source: Front Vet Sci. 2022 Jul 22;9:888207. doi: 10.3389/fvets.2022.888207 (PMC9354845; doi:10.3389/fvets.2022.888207)
Supplement: Supplementary file 1 [file Data_Sheet_1.ZIP › Supplementary file/miRDeep2 file.zip!/miRDeep2 file/Know_Structure/SPLP42A1/oar-mir-411b.pdf]

miRBase precursor : oar-mir-411b
Total read count : 2643
oar-miR-411b-5p read count : 2589
oar-miR-411b-3p read count : 52
remaining reads : 2

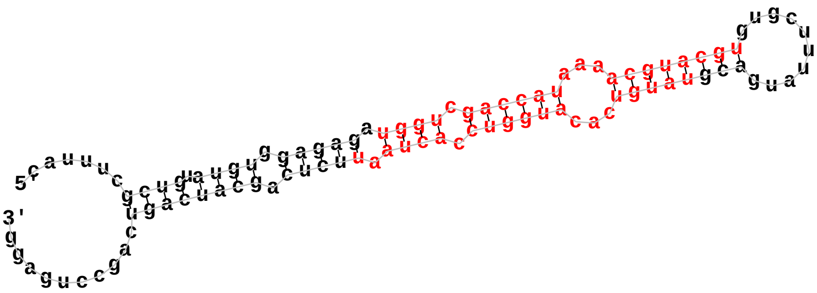

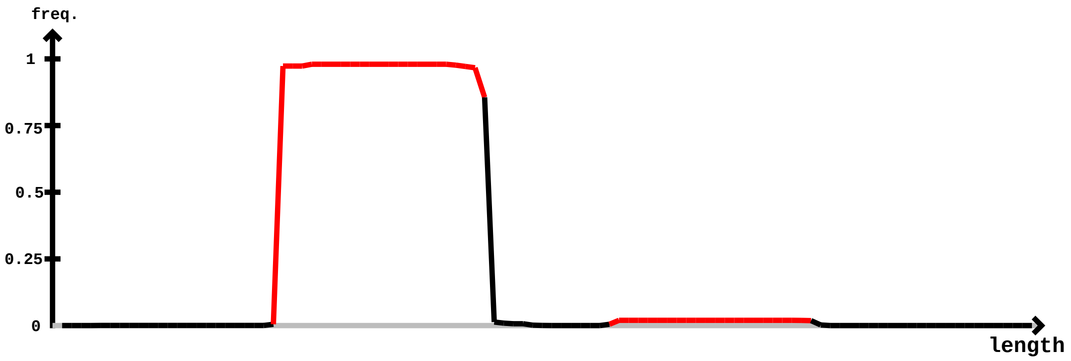

oar-miR-411b-3p

oar-miR-411b-5p

```
5'- cauuucgcuguauguggagagauggucgaccauaaaacguacgugugcuuuaugacguaugucacaugguccacuaauucucagcaucagucagccugagg  -3'    exp
    ......((((.((((.(((((.((((.((((((...((((((((..........))))))))...)))))).)))).))))).)))))))).........   reads   mm   sample
    ...uucgcuguauguggagaga...............................................................................   1       0    seq
    .....................auggucgaccauaaaacguac...........................................................   2       0    seq
    .....................auggucgaccauaaaacguacg..........................................................   9       0    seq
    .....................auggucgaccauaaaacguacgu.........................................................   1       0    seq
    ......................uggucgaccauaaaacgu.............................................................   9       0    seq
    ......................uggucgaccauaaaacgua............................................................   13      0    seq
    ......................uggucgaccauaaaacguac...........................................................   11      0    seq
    ......................uggucgaccauaaaacguacg..........................................................   284     0    seq
    ......................uggucgaccauaaaacguacgu.........................................................   2226    0    seq
    ......................uggucgaccauaaaacguacgug........................................................   11      0    seq
    ......................uggucgaccauaaaacguacgugu.......................................................   6       0    seq
    .........................ucgaccauaaaacguacgugugc.....................................................   14      0    seq
    .........................ucgaccauaaaacguacgugugcu....................................................   3       0    seq
    .........................ucgaccauaaaacguacgugugcuu...................................................   1       0    seq
    ........................................................guaugucacaugguccacuaa........................   2       0    seq
    ........................................................guaugucacaugguccacuaau.......................   10      0    seq
    ........................................................guaugucacaugguccacuaauu......................   1       0    seq
    .........................................................uaugucacaugguccacua.........................   1       0    seq
    .........................................................uaugucacaugguccacuaau.......................   33      0    seq
    .........................................................uaugucacaugguccacuaauu......................   5       0    seq
```
